# An Exploration of Barriers, Facilitators, and Suggestions for Improving Electronic Health Record Inbox-Related Usability: A Qualitative Analysis

**DOI:** 10.1001/jamanetworkopen.2019.12638

**Published:** 2019-10-04

**Authors:** Daniel R. Murphy, Traber D. Giardina, Tyler Satterly, Dean F. Sittig, Hardeep Singh

**Affiliations:** 1 Center for Innovations in Quality, Effectiveness and Safety, Michael E. DeBakey Veterans Affairs Medical Center, Houston, Texas; 2 Department of Medicine, Baylor College of Medicine, Houston, Texas; 3 School of Biomedical Informatics, University of Texas Health Science Center at Houston, Houston; 4 Center for Healthcare Quality and Safety, University of Texas Health Science Center at Houston, Houston

## Abstract

**Importance:**

Managing messages in the electronic health record (EHR) inbox consumes substantial amounts of physician time. Certain factors associated with inbox management, such as poor usability and excessive and unnecessary inbox messages, have been associated with physician burnout. Additionally, inbox design, usability, and workflows are associated with physicians’ situational awareness (ie, perception, comprehension, and projection of clinical status) and efficiency of processing EHR inbox messages. Understanding factors associated with inbox usability could improve future EHR inbox designs and workflows, thus reducing risk of burnout while improving patient safety.

**Objective:**

To determine barriers, facilitators, and suggestions associated with EHR inbox-related usability.

**Design, Setting, and Participants:**

This qualitative study included cognitive walkthroughs of EHR inbox management with 25 physicians (17 primary care physicians and 8 specialists) at 6 large health care organizations using 4 different EHR systems between May 6, 2015, and September 19, 2016. While processing EHR inbox messages, participants identified facilitators and barriers associated with EHR inbox situational awareness and processing efficiency and potential interventions to address such barriers. A qualitative analysis was performed on transcribed recordings using an inductive thematic approach with an 8-dimension sociotechnical model as a theoretical lens from May 6, 2015, to August 15, 2019.

**Results:**

The cognitive walkthroughs identified 60 barriers, 32 facilitators, and 28 suggestions for improving the EHR inbox. Emergent data fit within 5 major themes: message processing complexity, inbox interface design, cognitive load, team communication, and inbox message content. Within these themes, similar barriers were identified across sites, such as poor usability due the high numbers of clicks needed to accomplish actions. In certain instances, an identified facilitator at one site provided the exact solution needed to address a barrier identified at another site.

**Conclusions and Relevance:**

This qualitative study found that usability of the EHR inbox is often suboptimal and variable across sites, suggesting lack of shared best practices related to information management. Implementation of optimized design features and workflows will require EHR developers and health care organizations to collectively share this responsibility. Development of regional or national consortia to support collaborative sharing and implementation of EHR system best practices across EHR developers and health care organizations could also improve safety and efficiency and reduce physician burnout.

## Key Points

**Question:**

What factors are associated with physicians’ situational awareness and efficiency of processing messages delivered via electronic health record (EHR) inboxes?

**Findings:**

This qualitative analysis included interviews with 25 physicians in 6 health care organizations and identified 60 barriers and 32 facilitators associated with EHR inbox designs and workflows and 28 unique suggestions for improvement. Physicians suggested EHR developers and health care organizations reduce message processing complexity, simplify interface design, provide features to reduce physician cognitive load, facilitate care team communication, and streamline inbox message content.

**Meaning:**

This study’s findings included multifaceted recommendations associated with improving EHR inbox usability and workflow to improve safety and efficiency and reduce physician burnout.

## Teaser

This qualitative analysis using interviews with physicians determines barriers and facilitators in electronic health record inbox management and suggestions for improvement.

## Introduction

Physician burnout is increasing^1^ and has been associated with poor productivity,^2^ worse patient satisfaction,^3,4^ physician exhaustion and cognitive fatigue,^5^ decreased rapport with patients,^6^ delays in information processing,^7^ increased medical errors,^8^ and worse patient outcomes. Increasing workloads, often mediated by electronic health record (EHR) systems, increasing administrative burden, worsening work-life integration, poor clinic team structure,^9^ and erosion of autonomy have been suggested as causative factors for burnout.^10^ While most health care practitioners agree that EHR systems have improved communication and quality of care,^11^ the additional regulatory and administrative tasks that accompany EHR systems are associated with increasing physicians’ workloads and reductions in autonomy.^11,12^

Physicians spend approximately half of their clinic time on clerical and administrative tasks,^13,14^ including processing electronic messages delivered via EHR system–based inboxes. Such inboxes function similarly to email but deliver messages to clinicians, such as test results, messages from staff and other clinicians, medication refill requests, direct messages from patients via patient portals,^15,16,17^ and additional computer-generated messages.^18^ Management of the EHR inbox can add a substantial amount of time to physicians’ work lives. Primary care physicians spend between 49 and 85 minutes per day managing their inbox.^13,15,16^ Some inbox messages are irrelevant to the recipient and can reduce their ability to quickly identify important and timely information.^17^

While burnout and its association with EHR systems have received significant recent attention,^13,14,18,19,20^ the specific contribution of the EHR inbox has received limited evaluation, to our knowledge. A 2019 study^18^ suggested that excessive numbers of inbox messages, particularly those that are computer-generated rather than originating from patients, staff, or other clinicians, were associated with increased symptoms of burnout among physicians. While reducing the total number of messages sent to physicians may serve as one solution, many messages are important, and physicians often have unique and individualized preferences regarding what messages they want to receive. Improving EHR inbox designs and workflows could potentially allow physicians to safely and efficiently manage much of the workload. For instance, poor inbox usability (ie, the extent to which technology can be used efficiently, effectively, and satisfactorily^21^) can lead to inefficiencies that prolong the time clinicians take to process information, leading to frustration and exhaustion. Furthermore, these inefficiencies are associated with loss of situational awareness (SA)^22,23^ that may cause physicians to miss critical findings, such as abnormal test results.^19,24^ As a concept, SA was originally studied and applied in aviation, and it involves the ability to identify and understand information on 4 levels: (1) perception of elements in the environment, (2) comprehension of their meaning, (3) projection of their status in the near future, and (4) awareness of the best path to follow.^25^ Poor SA among health care practitioners has been associated with delays in care and diagnostic errors.^26,27^

As an initial step to improving EHR inbox designs and workflows for this study, we obtained physician input using cognitive walkthroughs to uncover barriers, facilitators, and suggestions for improvement that were associated with inbox usability, processing efficiency (ie, ability to process information quickly and without unnecessary steps), and SA at the point of care. Our overall goal was to inform strategies for better EHR inbox designs and optimized workflows to minimize clinician burden and improve safety.

## Methods

This study was approved by the institutional review board of Baylor College of Medicine. Verbal informed consent was obtained from all participants. Methods and findings are reported based on Standards for Reporting Qualitative Research (SRQR) reporting guidelines.

We recruited physicians at 6 large health care organizations (HCOs) in southeast Texas between May 6, 2015, and September 19, 2016. We contacted the leadership at each HCO to disseminate information about our study to all physicians via email or at faculty meetings. We enrolled the first 25 physicians who were interested and provided study details via email or telephone. If the physicians agreed to participate, we set up in-person meetings at their clinics or administrative offices. Prior to the meeting, we informed participants that their deidentified perspectives would be used for research publication, and we obtained verbal informed consent for participation and to audio record discussions. No compensation was provided to participants.

After collecting information on the physician’s specialty and which EHR system they used, one of us (D.R.M.) with a background in clinical medicine, informatics, process improvement, and qualitative research performed an adapted cognitive walkthrough with each physician. Cognitive walkthrough is a human factors method used to understand system usability associated with the performance of each step of a task and is especially well suited to identifying real or perceived barriers to accomplishing a task.^28,29^ While cognitive walkthroughs are typically reserved for interface designs, we adapted this method to obtain findings beyond the user interface and to explore barriers to inbox message processing, including inbox design and related workflows outside of the EHR system. Thus, we included broader sociotechnical dimensions that are associated with message processing, such as workflow, staffing and personnel, and organizational policy and culture.^30^ Physicians were asked to indicate when a workflow barrier was encountered, suggest a potential improvement to that barrier, and indicate when a feature was encountered that particularly facilitated the workflow. *Situational awareness* was defined as the physician’s’ ability to understand a patient’s clinical status sufficiently well to determine the next steps to take. Physicians were free to discuss any type of message they received. For anonymity, individual sites were identified by letters (ie, A-F) and individual physicians were identified by numbers (ie, 1-25).

### Qualitative Analysis

Audio recordings were transcribed for analysis. Two of us (D.R.M. and T.S.) with expertise in clinical medicine, informatics, quality and safety, human factors, and qualitative research served as coders and reviewers. The coders familiarized themselves with all of the data and then coded transcripts using Atlas.ti data analysis software version 7.5 (ATLAS.ti Scientific Software Development). Analysis was supervised by 2 of us (H.S. and T.D.G.) with extensive experience performing qualitative research. The individuals coding the data independently identified each mention of a facilitator, barrier, or suggestion for improvement of the inbox design or workflow that was associated with SA or efficiency. To identify statements associated with SA, coders identified statements regarding the perception or comprehension of information about their patients, projection of future status, or decisions about next steps to take. Statements associated with efficiency were identified via mention of activities taking longer or shorter amounts of time or with additional or fewer steps than expected. After an initial 5 transcripts were coded, the coders met to reconcile similar codes into a common codebook and discuss and incorporate any emergent codes. This process was repeated each week until all of the transcripts were coded.

Once coding was complete, the reviewers used thematic analysis^31,32^ to group codes that conveyed similar barriers, facilitators, and suggestions for improvement into increasingly higher-level themes. The transcripts were then merged, and discrepancies in themes between coders were discussed by both coders and reconciled by consensus prior to final analysis. To facilitate a comprehensive view of the data, we used the consensus methods to incorporate the diverse professional backgrounds of both reviewers. Finally, we organized codes between and across sites into meaningful conceptual relationships using an 8-dimension sociotechnical model for health information technology^30^ as a theoretical framework to identify relationships among themes.

## Results

We recruited a total of 25 participants from 6 different health systems. Table 1 presents site and physician characteristics. Participants included 17 primary care physicians (internal medicine and family medicine) and 8 specialists (oncology, cardiology, psychology, otolaryngology, pulmonary, and infectious disease).

**Table 1.** Site and Physician Characteristics

Characteristic	Site						Total
	A	B	C	D	E	F	
Electronic health record system (version)	VistA/CPRS (1.0.31)	Epic (2012)	Epic (2015)	Epic (2012)	GE Centricity (9.5)	AllScripts (11.4)	
Physicians, No.							
Primary care	3	5	5	0	2	2	17
Specialist	2	2	0	3	0	1	8
Total	5	7	5	3	2	3	25

Cognitive walkthroughs required a mean (SD) of 33.0 (15.3) minutes. Qualitative analysis of the 25 transcripts resulted in identification of 60 barriers and 32 facilitators (eAppendix in the Supplement). Twenty-eight types of suggestions for improvement were identified (Table 2). Themes were grouped into 5 categories: message processing complexity, inbox interface design, cognitive load, team communication, and message content.

**Table 2.** Physician-Recommended Changes to Improve Situational Awareness and Efficiency Associated With Electronic Health Record (EHR) Inbox Management

Theme	Recommendation
Message processing complexity	Ensure EHR-based workflows match clinical workflows Simplify workflow by reducing the number of mouse clicks to accomplish an action Allow templated text of common test results interpretations for patients Provide contextual information (eg, trends) that is easily accessible form the inbox Ensure related results (eg, all results in a panel) are presented together and in a manner physicians are accustomed to Ensure that message subject line matches the full contents of the message
Inbox interface design	Highlight abnormalities or other deviations from normal to make them more salient Allow test results to be quickly trended over time Hide interface elements that are irrelevant, distracting, or duplicated When possible, allow physicians to customize displays to their needs Ensure that interface elements are easy to understand and avoid technical terminology Allow features to prioritize messages and ensure priority messages are easily visible Deliver messages to a single inbox or ensure that rules for delivery to different inboxes are clear and practical
Cognitive load	Allow customizable reminders or to-do lists that remind physicians to take action for a particular patient at a future date Provide ability to assign priority to messages and enable sorting by priority to triage work Allow messages to have added comments or tags to facilitate subsequent review Allow flagging, sorting, and filtering of messages to enable prioritization and triaging Allow new messages to be easily distinguished from previously read messages
Team communication	Prevent messages from disappearing until the physician explicitly indicates that action is desired, such as via a *complete* button Allow read receipts to ensure closed-loop communication Provide out-of-office messages to indicate when an inbox is not being actively monitored Provide a way to manually forward inbox messages to others Allow physicians to automatically receive or otherwise review messages transmitted to other physicians whose patients they may be temporarily caring for Incorporate staff into the message triaging process rather than sending all messages to physicians Allow tasks to be distributed among clinical team members
Message content	Reduce transmission of messages that do not affect care Educate staff to avoid transmission of messages to clinicians when the message does not affect care the clinicians provide Prevent duplicate messages

### Message Processing Complexity

Physicians indicated that EHR inbox features and workflows did not always match clinical processes and frequently required more steps than physicians felt should be necessary. In many cases, taking follow-up action in response to messages required additional steps perceived as unnecessary, such as the need to create new encounters because the EHR system would not permit physicians to use existing encounters that had been closed. For example, physician 10 reported, “They’ll send me a message back that just says, ‘Patient wants referral to physical therapy,’ but I can’t use that encounter to order the referral.” In general, physicians preferred methods that could streamline message processing and required fewer clicks, such as the comment by physician 24, “But still that’s a lot of clicks. I mean, if you look at all the messages that you have, how many clicks is that for each one?”

Participants reported that features that simplified workflow, such as the use of quick notes, which facilitated documentation from the inbox, or results interpretation letter templates, helped streamline message processing. In many cases, physicians promoted methods that reduced clicks, even if they saved small numbers of clicks during each use. For example, physician 20 reported about one method, “…It just saves about 5 clicks,” and physician 1 stated, “…So that’s just a nice way to save a few clicks.”

Similarly, physicians reported that the presentation of information in the inbox did not always support SA or efficiency because of a lack of contextual information. Information was often presented in ways that were unexpected or not intuitive. For example, physicians reported difficulty in interpreting results when they were sorted alphabetically rather than grouped into related tests (eg, individual laboratory panel results grouped together) or 2 or more separate tests were grouped within a single message (eg, both magnetic resonance and radiographic imaging results grouped into an “Imaging result” message).

Physicians recommended new EHR features that would provide rapid access to contextual information about the message that they were currently processing. This would allow them to quickly scan key contextual clinical factors to make decisions. Examples of relevant contextual information included upcoming appointments, laboratory test trends, active problem lists, current medications, their own last progress notes, patients’ contact information, assigned primary care physicians, and certain risk calculations (eg, cardiovascular risk) either within or easily accessible from the inbox. In certain cases, physicians recommended that specific information be automatically shown based on the message content. For example, physician 12 stated, “I’m refilling cholesterol medicine. I need to know what was their last cholesterol value. So that value should somehow, either the staff should prepopulate it, or this type of drug should trigger it. Something should happen so that now I don’t have to look for it.”

In certain EHR systems, this contextual information was available, and physicians overwhelmingly reported benefits of this feature for both message processing efficiency and SA. Physician 20 stated, “It used to be that [the EHR system] just gave me results from the most recent labs, and there were no trends, and so it would take a long time for me to open up their actual chart and see what their trends are. For example, if someone’s creatinine is like 1.5, then I’m like ‘Is that new or is that old?’ and then I have to go back and look. Now, they trend it for you.” Physician 14 stated, “You don’t have to go into the chart, but there’s a button you can push to pull up the medication list and the problem list.… It’s helpful.” Physician 20 further stated, “And what I also like about this feature is that the patient’s phone number is here [in the inbox]. I know who the primary care doctor is. It’s all listed here. The phone number is important because if there is an abnormal lab, …I have the phone number here, and I call right away instead of having to open up the chart and figure out what the patient’s phone number is.”

In some cases, providers reported inability to accomplish actions that they expected the interface to allow them to do. As an example, physician 3 reported “[This alert] doesn’t even have a name on it, but it shows on my list because the patient is assigned to me. I don’t need to sign it… but it won’t go away. It’s been there since [6 months ago].”

### Inbox Interface Design

Physicians described several features of the interface design that were perceived to facilitate SA. Certain inbox interface layouts improved organization of information and allowed data to be more quickly and easily interpreted, such as a description from physician 17 of the EHR system highlighting abnormal test results: “This is a lab result. Then I look at the trend because I’m interested in it, and I see that it’s fine and that it’s normal. Then, I come here. I’m going to scroll down… I see one abnormal result has been highlighted, and I like that, that it’s yellow, so it really flags me to pay attention to it.”

Other recommendations included limiting clutter of information on the interface, such as hiding message folders that are empty. Physician 16 described the clutter, “You see how many categories there are. There are multiple categories, although some of them are not used that much, like *letter queue*. I don’t even know what that’s for. *Patient questionnaire*, I don’t know what that’s for. I never use that.”

Another suggestion was allowing physicians to customize displays to hide interface items that are irrelevant, distracting, or duplicated. As an example, physician 10 described, “So, there are a lot of columns that the individual user…can quickly hide, get rid of, and get rid of a bunch of fluff.”

Physicians also identified several barriers to SA, such as interfaces that included unclear icons, error messages with technical jargon, or confusing layouts that resulted in confusion. Physician 18 stated, “And as far as this *delegate* feature, I don’t know what that means. I mean, I know what the word *delegate* means, but I don’t know what it means here.”

Other physicians noted that the interface did not facilitate efficient prioritization of messages and offered only limited message filtering or flagging capabilities. Physician 3 stated, “I wish there was a way that I could save [the message] as a high [priority message]…. Let’s say I… started to work on [a message] and didn’t make my call. Somebody came in urgently, so I had to stop and just make it as an addendum as a reminder later. But now it gets under these things. I don’t know which one is the high [priority message] in this one.”

Physicians at one site reported that their EHR system used 2 separate inboxes but that it was unclear which messages would be routed to each inbox. They also stated that they may be less likely to check one of the inboxes frequently, increasing the likelihood of delays in responding to messages. Physician 18 stated, “Sometimes that lab result will only land in [one inbox]. Sometimes it’ll land only in my [other inbox]. Sometimes it’ll land in both.”

### Cognitive Load

Physicians described software features that were positively or negatively associated with cognitive load (ie, the amount of working memory required by clinicians to complete a task). They identified several facilitators that provided the ability to add comments and flags to messages to serve as reminders when messages could not be processed immediately. They additionally used a *remind me* feature to send a message to themselves either immediately (to serve as a to-do list) or at a future date, allowing them to reduce the quantity of messages present in their inbox. Physician 10 described the *remind me* feature as, “This is [the] *remind me* [feature], and I, personally, put mine right in the center of our [EHR] toolbar because I want it quick and fast when I’m in the middle of a visit. So, this one, I saw him yesterday. He needs a preop letter. I don’t have time to hand him the preop letter in the middle of my workday. I may do it this weekend, but I need it on my to-do list. If it’s not in front of my face, I’m not going to do it. So, *remind me* is purely what I need to do.”

Conversely, physicians whose EHR inboxes had no such features mentioned that lack of reminder capabilities increased their cognitive load, requiring them to use other means outside of the EHR system to remember to take future actions. These physicians suggested adding the capability to set date-based reminders or create a to-do list in the inbox, as well as the capability to create general reminders not necessarily attached to a specific patient. Physician 20 stated, “I would love to have a place where I can have like a sticky note or a ‘things to do’ because I forget.”

Several of the suggestions were associated with improving prioritization features. Physicians suggested that methods should exist to flag or filter messages; reorganize, group, and sort messages; or triage messages and assign priority. For example, physician 3 stated, “Because we like to triage. We are trained to triage things by order of importance, and, again, every line has the same order of importance, and everything is tagged as red flags. We need more flags.” Similarly, physician 18 stated, “If there was some way that I could annotate the tasks without sending my own tasks, like reading the original one and sending myself my own tasks. If I could just right-click on something and write a little note about it.” When the interviewer (D.R.M.) asked, “Like a little *comments* type of thing?” physician 18 went on, “Yeah, so I could say, ‘Okay, I’m waiting on this one.’ That’s why I haven’t processed it, or I’m debating about whether to do this or that. I’ll present my options and make a decision there. So yeah, that would be nice.”

Physicians also recommended features to distinguish new messages from read messages in the inbox. Another feature physicians suggested was one to differentiate messages regarding patients they were caring for temporarily (eg, while a colleague is out of the office) from messages related to their own patients.

### Team Communication

The study team found several concerns associated with communication with other members of the clinical team, including staff and other clinicians. As a facilitator to inbox management efficiency, physicians reported the need for more thoughtful distribution of tasks to appropriate clinical team members who could triage messages. These included using staff protocols to refill certain medications when specific criteria are met or having staff preload relevant information or orders (eg, patient-requested screening tests or refills) into an encounter prior to routing it to a physician. Physicians reported that this allowed them to quickly sign or decline orders rather than spending time entering the order themselves. Physician 16 gave an example, “The nurse, if they’re good, they’ll already pend the [patient-requested] orders for me.”

There were several suggestions to improve communication between clinicians by ensuring receipt of messages by the intended recipient. Physicians suggested adding features to determine if a sent message had been opened by the recipient (eg, read receipts, message opening timestamps), and to indicate to anyone attempting to send a message that a physician was out of the office. They also advocated for staff to help triage messages and handle things that were within their scope of practice rather than sending all messages directly to the physician. Physician 24 said, “I kind of wish [patient portal messages] would [be triaged by staff]. Sometimes they are silly things that I don’t need to address. So, I got this one… this one certainly could have been triaged. It would have been nice if it was.”

### Message Content

Additionally, physicians discussed information conveyed by messages, such as low-value messages and duplicate messages, that do not affect patient care. Both types of messages were deemed to take up space in the inbox, distract from more important messages, and thereby decrease efficiency and SA. Physician 3 gave an example of low-value messages as, “Here’s a completed order, so all that means is they got a referral to ENT and they went to ENT. Okay. I don’t really care. I mean I can see how someone would say well, let’s just go ahead and give [physicians] that information just in case. Okay, but I’ve got 6 folders of just-in-case stuff that means nothing to me.... I just end up [clicking] *select all*, *done*, and then get rid of them, because I’ve got too much stuff to go through individually. They’re trying to give me everything, and as a consequence, I don’t have time to click through everything. Show me the important stuff, and then we’ll go through it.” Physician 3 also described an example of duplicate messages as, “This is the annoying part of x-rays; let me explain to you why. You saw the other 3 up here? They were all for the same person, same testing. Exact same test: CT abdomen pelvis. I get 3 because the way they process it; they process it in 3 reconstructions, so all 3 of them [generate a separate message].”

To solve these issues, physicians suggested reducing messages with little clinical relevance, such as only sending messages when clinical processes are not proceeding as planned. Physician 20 gave an example as, “I guess that if he didn’t send me a confirmation, I would have been okay with that. I trust him. I talked to him in person.… When it doesn’t go as planned, that’s when I want to know. I don’t need to be told about everything.”

Physicians also suggested limiting content to a format that efficiently relayed information. In the context of referrals, they suggested sending a summary of patients who had been scheduled instead of sending a message for each patient individually.

### Association of Themes With Efficiency and SA

The themes we identified were associated with 5 sociotechnical dimensions: hardware and software (cognitive load theme, specifically the presence of EHR features that assisted physicians in remembering information for future action), content (message content), user interface (inbox interface design), personnel (team communication), and communication and workflow (message processing complexity and team communication). All the themes were associated with physician efficiency. The cognitive load, message content, and inbox interface design themes were identified as being associated with SA. The Figure presents the associations among these concepts.

**Figure.**
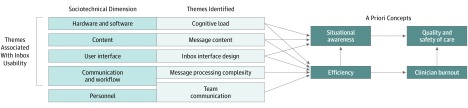
Associations of Themes Identified With Sociotechnical Dimensions, Situational Awareness, Efficiency, Burnout, Quality, and Safety

## Discussion

We identified several design features that were associated with physicians’ EHR inbox management efficiency and SA. Themes included message processing complexity, inbox interface design, cognitive load, team communication, and message content, all of which could serve as targets for future improvement efforts by software designers, health care organizations, and researchers. While some of these concepts are similar to those identified in other aspects of EHR-enabled health care, they have been inadequately addressed thus far and the inbox has mostly escaped scrutiny, to our knowledge. Factors such as excessive message processing complexity and lack of features to reduce cognitive load may be associated with reduced efficiency and situational awareness in inbox management. Thus, usability of EHR inboxes has made minimal progress in the past 20 years since EHR systems have been widely implemented.

Several concerns identified were associated with poor efficiency of message processing owing to high numbers of clicks needed to accomplish actions. Physicians remarked that individual actions taken in poorly designed EHR inbox interfaces each contributed a small number of unnecessary clicks but, when multiplied by the large number of messages physicians received each day, significantly affected their workloads.^15^ Examples of the inefficiencies included activities for opening and searching the EHR for different pieces of contextual information that may be located in different areas of the EHR and that are needed when processing messages (eg, checking the last lipid panel results when refilling a cholesterol medication). This may explain why physicians preferred to process messages directly within the inbox when possible and recommended certain contextual information be presented in the inbox along with the message. Excessive clicking is a common complaint by physicians about EHR systems and has been associated with burnout.^33,34^ Thus, similar to other aspects of EHR system use, efforts are needed to ensure that EHR inboxes are designed and configured to minimize unnecessary clicking.

Our findings add to previously described difficulties regarding physician experiences with EHR inboxes,^1,5,13,15,34,35^ including our 2019 study^36^ on physicians’ best practices for managing messages in the EHR inbox. This study expands on that knowledge by identifying specific barriers and facilitating features for EHR inbox designs and workflows, providing targets for improving usability. Themes identified that affect the technical (rather than social) aspects of EHR inbox message processing correlate with other recommendations for user-centered design. For example, within our theme of message processing complexity, recommendations from physicians that EHR-based workflows should match clinical workflows directly relates to Neilsen’s usability heuristics recommending “match between system and real world,” while suggestions for macros, templated text, and preference lists relate to “flexibility and efficiency of use.”^37^ Additionally, our findings help to identify aspects of EHR inbox designs and workflows that are associated with burnout, quality, and patient safety (Figure).

We provided specific recommendations for future studies involving diverse stakeholders, including EHR vendors, HCOs, and researchers (Table 2). Our findings highlighted several interrelated clinical and team-based processes in inbox management associated with efficiency and SA but which neither EHR vendors nor HCOs can address on their own (eg, ensuring the clinic workflows match EHR workflows). Thus, EHR vendors and HCOs both bear responsibility for efficiency and SA issues in EHR system use. They will need to work collaboratively to improve the safety and efficiency of inbox management and disseminate best practices to other organizations. Given that EHR vendors already work with different HCOs, they are in an optimal position to serve as hubs for collecting, implementing, and diffusing best practices. Furthermore, this study identified specific recommendations for future study, such as conducting quantitative research to assess the feasibility and expected effect of the recommendations, assessing the actual effect of specific recommendations on SA and efficiency, and discerning differences in needs between primary and specialty care.

Several facilitators provided potential direct solutions to the identified barriers, supporting their implementation given modern technological capabilities. This occurred both within the same EHR system implemented at different sites and across different EHR systems. We identified one instance within the same EHR product where a specific feature was implemented differently at different sites. Specifically, a participant reported as a barrier the need for paper notes as reminders to follow up on future activities (eg, remembering to check whether a repeated test has been completed), while a clinician at a different site using the same EHR system reported the benefits of using a future reminder function built directly into the EHR system’s inbox. This suggests that physicians and organizations may have disparate features activated or be unaware of helpful features and therefore might use EHR inboxes differently. Several instances were identified in which different EHR systems contained facilitators that directly addressed barriers in another site’s EHR system. For example, some physicians recommended certain relevant information (eg, prior results, medication lists, patient contact information) be easily accessible from the inbox itself without additional steps needed to enter the full EHR to access it, while physicians at other sites indicated that similar features were already available within the inbox. Some physicians requested a method to access previously completed messages, while physicians at other sites indicated the presence of archives within the EHR system that allowed retrieval of such messages. Furthermore, physicians whose EHR system contained 2 separate inboxes articulated concerns about not knowing which inbox to expect a message in; such concerns would likely not be present in single-inbox systems. However, none of the participants mentioned a specific method to share best practices with their colleagues using the same EHR system in different organizations, and such sharing is even less likely to occur between organizations with different EHR vendors. Policy makers should stimulate the development of regional or national consortia to share best practices to improve EHR system efficiency and SA, which could drive more rapid advancement of better EHR designs and organizational processes.

### Limitations

This study had several limitations. First, this study was performed at 6 sites using 4 different EHR systems; thus, our findings do not represent the wide variation in EHR systems and workflows that exists, including in inpatient settings. Similarly, the small sample of specialty physicians limited comparison of inbox needs between primary and specialty care. However, this exploratory study could serve a basis for future work to understand how identified barriers and facilitators are associated with inbox management. Second, the findings were based on physician discussion, and actual efficiency and SA were not directly measured. However, our study may serve as a basis for future work to test the effect of specific interventions. Third, participants may have failed to disclose barriers owing to recall bias or response bias, and responses may have been affected by physicians’ knowledge of EHR system features, which may not have been complete given the complexity of EHR software. Such limitations are present in most qualitative studies and are partially mitigated by interviewing multiple participants at each site.

## Conclusions

In conclusion, this qualitative study identified barriers, facilitators, and suggestions associated with physicians’ SA and efficiency of processing messages in the EHR inbox. Emergent themes of message processing complexity, inbox interface design, cognitive load, team communication, and message content could improve future EHR inbox design and workflows. However, such efforts to improve usability would require a shared responsibility between EHR developers and health care organizations as well as sharing of design features and workflows, potentially via development of regional or national consortia to share best practices. Implementation of these multifaceted recommendations to improve safety and efficiency and reduce physician burnout would require a strong collaborative effort between EHR developers and health care organizations.

## Notes

Supplement. **eAppendix.** Codebook of Barriers and Facilitators
