# Supplementary material for: An Exploration of Barriers, Facilitators, and Suggestions for Improving Electronic Health Record Inbox-Related Usability: A Qualitative Analysis
Source: JAMA Netw Open. 2019 Oct 4;2(10):e1912638. doi: 10.1001/jamanetworkopen.2019.12638 (PMC6784746; doi:10.1001/jamanetworkopen.2019.12638)
Supplement: Supplement. — eAppendix. Codebook of Barriers and Facilitators [file jamanetwopen-2-e1912638-s001.pdf]

Supplementary Online Content

Murphy DR, Giardina TD, Satterly T, Sittig DF, Singh H. An exploration of barriers, facilitators, and suggestions for improving electronic health record inbox-related usability: a qualitative analysis. *JAMA Netw Open*. 2019;2(10):e1912638. doi:10.1001/jamanetworkopen.2019.12638

eAppendix. Codebook of Barriers and Facilitators

This supplementary material has been provided by the authors to give readers additional information about their work.

© 2019 Murphy DR et al. *JAMA Network Open.*

eAppendix. Codebook of Barriers and Facilitators

**Barriers**	
1	Additional steps needed to determine who PCP is
2	Cannot remove message
3	Cannot route note to PCP if no PCP assigned
4	Cannot send fax without being in encounter
5	Commonly used buttons far away from each other
6	Different messages arrive in different inboxes, and secondary inbox might not be checked as regularly
7	Difficult to distinguish similar message types from each other in inbox
8	Difficult to see message sender
9	Duplicate Messages
10	EHR does not support self-reminder functionality
11	EHR slow to load data
12	Entering "future reminder" date for messages is on a different screen
13	Error messages are unclear
14	Going into chart is less efficient than processing form inbox
15	Info-only messages do not link to relevant portion of record
16	Lab results displayed in unsorted or unusual sequence
17	Lack of context for messages without entering chart
18	Layout is confusing
19	Low Value Messages
20	Merging of unrelated reports in a single message
21	Message threads/replies are not always in order (difficult to follow discussion)
22	Message to patient is added to results inappropriately
23	Messages disappear before provider intends
24	Messages to patients do not show actual sender, just "office of..."
25	Multiple Locations for Messages
26	Multiple steps to get to release result screen
27	Must set up a list of names to efficiently forward messages
28	Need to click "more" to see other buttons/features that don’t fit on menu
29	Need to document message to patient and similar/same message in another box for staff to act
30	Need to enter the chart for recurrent and straightforward actions
31	Need to know recipients’ names (no way to send to department pool)
32	Need to start letter over if results not signed first
33	No ability to get to images from radiology results without entering the chart
34	No indication of message priority level or flag
35	No way to decline/reassign message responsibility
36	No way to tell if provider is the only one receiving a result
37	Not able to compare prior faxes to see if they are duplicates or in series
38	Not able to convert messages/result notes into encounters for placing orders

© 2019 Murphy DR et al. *JAMA Network Open.*

39	Not able to get directly into patient's chart via inbox
40	Not able to see messages delivered to residents being supervised
41	Not able to sort messages
42	Not able to tell meaning of message from the inbox
43	Not able to use quick actions in releasing results to patient portal
44	Not clear what icons on buttons mean
45	Patient messages go to nurse pool instead of directly to provider
46	Physicians forget to check second inbox
47	Process to get to orders is long
48	Related and commonly used buttons far from each other
49	Some messages cannot be ever retrieved after deletion
50	Technical problems prevent local printer from working
51	Two places to type result interpretations, not clear which patients prefer or see
52	Unable to see another physician's inbox when covering
53	Unable to see other related information at the same time during decision making
54	Unclear EHR terminology
55	Unclear rules that govern where message will arrive
56	Unclear why similar EHR tools lead to different outcomes
57	Unnecessary steps needed to send messages
58	Unnecessary steps to document results
59	Unused categories clutter inbox
60	When placing orders (e.g. radiology), if they are not routed, they do not get scheduled

**Facilitators**	
1	Ability to add personal note to a message
2	Ability to change priority of a message
3	Ability to easily see own clinic schedule from the inbox
4	Ability to hide irrelevant/distracting/duplicate columns in inbox window
5	Ability to quickly address routine issues from within the inbox
6	Ability to release several laboratory results at once
7	Ability to review all results at once (instead of individually)
8	Ability to see if task is still on another person's list
9	Ability to see last wellness visit from the inbox
10	Ability to see next appointment from the inbox
11	Ability to see patient's phone number from the inbox
12	Ability to see PCP name directly in the inbox
13	Ability to see that there are X out of Y results ready
14	Ability to see trends directly in the inbox
15	Ability to use message-specific quick actions (avoid clutter of buttons irrelevant to message type)
16	Abnormal results are highlighted
17	All messages go to the same inbox

© 2019 Murphy DR et al. *JAMA Network Open.*

18	Buttons for commonly used quick actions
19	Clicking on Inbox Notification button takes directly to folder with high-priority message
20	Methods to create future self-reminders
21	Nurses have access to other physicians’ inboxes
22	Nursing protocols to refill specific medications if certain criteria met
23	Option for free-text header on lab results
24	Patient portal status easily visible from the inbox
25	Quick access to medication list from the inbox
26	Quick access to problem list from the inbox
27	Quick access to your last note from the inbox
28	Similar workflow for releasing test results to the patient portal and for generating a results letter to be mailed
29	Staff research and pre-load information and orders prior to sending messages
30	Use of "quick notes" allow documentation to occur within the inbox
31	Use of out of office feature
32	Use of templated result interpretations letters

© 2019 Murphy DR et al. *JAMA Network Open.*
